# Hydrogen and Oxygen Stable Isotope Fractionation in Body Fluid Compartments of Dairy Cattle According to Season, Farm, Breed, and Reproductive Stage

**DOI:** 10.1371/journal.pone.0127391

**Published:** 2015-05-21

**Authors:** Fabio Abeni, Francesca Petrera, Maurizio Capelletti, Aldo Dal Prà, Luana Bontempo, Agostino Tonon, Federica Camin

**Affiliations:** 1 Consiglio per la Ricerca e la Sperimentazione in Agricoltura, Centro di Ricerca per Produzioni Foraggere e Lattiero-casearie, via Porcellasco 7, 26100 Cremona, Italy; 2 Fondazione Edmund Mach—Centro di Ricerca Istituto Agrario San Michele all’Adige—Dipartimento Qualità Alimentare e Nutrizione, via E. Mach 1, 38010 San Michele all’Adige (TN), Italy; NYIT College of Osteopathic Medicine, UNITED STATES

## Abstract

Environmental temperature affects water turnover and isotope fractionation by causing water evaporation from the body in mammals. This may lead to rearrangement of the water stable isotope equilibrium in body fluids. We propose an approach to detect possible variations in the isotope ratio in different body fluids on the basis of different homoeothermic adaptations in varying reproductive stages. Three different reproductive stages (pregnant heifer, primiparous lactating cow, and pluriparous lactating cow) of two dairy cattle breeds (Italian Friesian and Modenese) were studied in winter and summer. Blood plasma, urine, faecal water, and milk were sampled and the isotope ratios of H (^2^H/^1^H) and O (^18^O/^16^O) were determined. Deuterium excess and isotope-fractionation factors were calculated for each passage from plasma to faeces, urine and milk. The effects of the season, reproductive stages and breed on *δ* ^2^H and *δ* ^18^O were significant in all the fluids, with few exceptions. Deuterium excess was affected by season in all the analysed fluids. The correlations between water isotope measurements in bovine body fluids ranged between 0.6936 (urine-milk) and 0.7848 (urine-plasma) for *δ* ^2^H, and between 0.8705 (urine-milk) and 0.9602 (plasma-milk) for *δ* ^18^O. The increase in both isotopic δ values in all body fluids during summer is representative of a condition in which fractionation took place as a consequence of a different ratio between ingested and excreted water, which leads to an increased presence of the heavy isotopes. The different body water turnover between adult lactating cattle and non-lactating heifers was confirmed by the higher isotopic *δ* for the latter, with a shift in the isotopic equilibrium towards values more distant from those of drinking water.

## Introduction

Water is the most common molecule in the body of vertebrates and in homeotherms it represents an important heat carrier for the regulation of thermal exchanges [1], as it allows the transfer of a large amount of heat in a small “carrier” volume [2]. This is particularly important in the case of high-producing dairy ruminants because their physiological adaptation is related to their energy metabolism, even when related to their metabolic weight (**BW** ^**0.75**^). In lactating dairy cows, water loss (output) occurs in various forms, from evaporation for thermoregulation [1, 3–4] as well as the production of milk, urine, faeces [2] and salivary secretion [5], whereas the main sources (input) of water include drinking, feed, and metabolic (oxidation) water [2]. Water intake is affected by dry matter intake (**DMI**), sodium intake, milk production, minimum environmental temperature and the water content of feed [4, 6]. Furthermore, in thermo-neutral conditions, salivary secretion is related to DMI, neutral detergent fiber (**NDF**) intake and the average particle size of the meal [7]. The total body water (**TBW**) of a lactating dairy cow weighing 640 kg could be estimated to be 422 L [8], and its biological half-life was calculated to range between 2.9 and 7.5 [9–10] days. In dairy cattle (and other categories), heat stress perceived by the animal (and therefore activation of the thermo-regulating response) is determined not only by air temperature (**Ta**) which heavily affects respiratory water loss [3], but also by air relative humidity (**RH**), solar radiation and wind speed near the animal [11]. The best applied summarising heat stress index is the temperature humidity index (**THI**), as reported by Dikmen and Hansen [12].

In the lactating cow, in thermoneutral conditions, water excreted by milk yield corresponds to approximately one third of the amount of water drunk [9, 13]. During heat stress periods, in environmental situations present in Italy and, more generally in southern Europe, there is an increase in the water requirement due to thermoregulation, which is the result of increased demand due to evaporation as a result of heat dissipation [3, 14]. This leads to a shift in the balance between total water intake (**TWI**) and its partition between body compartments in comparison to the cold season. From published results [13], we calculated that the water excreted through evaporation shifted from 17.2% to 28.8% of total excreted water in lactating cows, and from 26.4% to 42.0% of total excreted water in dry cows changing from a thermoneutral to a heat stress condition. This shift may imply an increase in fractionation of the body water pool.

West summarised some important points about the relationship between genetics and heat stress in dairy cattle, reminding us that the ability to maintain body temperature is heritable through characteristics including sweating competence, body tissue resistance, coat structure and colour [15]. However, he also reminded us that in the *Bos taurus*, the increased ability for thermoregulation is generally linked to a reduction in energy metabolism [15, 16]. Additionally, the sweating response was shown to be negatively correlated with metabolic rate and this may impair selection policy, which wishes to combine heat adaptation and milk yield potential traits in cattle, considering that genetic correlation (r_g_) between production and heat tolerance is approximately -0.3 [15, 16]. It is therefore possible that two breeds differing strongly in terms of their genetic background for milk production may differ in terms of their evaporative response to heat stress. According to the general physiochemical rules reported by Fry, this evaporation process is expected to be an isotopic fractionation point of the mammals’ TBW [17]. Thus, the two breeds should also have different water isotope fractionation in body fluids, which is related to evaporation, as vapour is more depleted in heavy isotopes than other body fluids [17, 18].

Today, the meaning of the change in the isotope composition of body water in human fluids is not fully clear, but growing interest is related to the possible relationships with water homeostasis [19] and kidney function [20].

The use of stable isotope analysis to assess the origin of dairy products is a method of growing interest in terms of discovering possible commercial fraud [21]. An increasing number of papers have studied the characterisation of milk and its relationship with the diet fed to cows, using carbon (**C**), nitrogen (**N**), oxygen (**O**) and hydrogen (**H**) stable isotope ratios determined in animal feed and milk or some of its fractions [22]. With this approach a series of observations on *δ* ^**2**^ **H** and *δ* ^**18**^ **O** values in milk and the factors probably affecting them were suggested. No demonstrated relationship was found between *δ* ^**18**^O and *δ* ^2^H in milk and an increase in the maize percentage in cattle diet [22]. In contrast, a relationship between *δ* ^18^O in milk water and the season was reported by Kornexl et al. [23], due to seasonal changes in the *δ* ^18^O of forage plants, as well as in the body of the animal, linked to evapotranspiration [23]. More recently, *δ* ^2^H and *δ* ^18^O stable isotopes in milk were used to detect its geographical origin, due to the relationship between the isotopic signature of milk and that of the drinking water of regions located at different latitudes and/or altitudes [24, 25]. Despite the potential of the ‘isotopic’ approach, it is necessary to consider possible sources of variation related to isotope fractionation that could affect the success of relating body water stable isotope conditions to those of the drinking water in a specific area over time, as already reported by Bryant and Froelich for O in different mammals [18].

The study of body water partitioning in dairy cattle was first investigated with a water stable isotope approach using the deuterium oxide method [10]. Later, Bryant and Froelich [18] set up the study of O isotopic fractionation in the body water of large mammals as a result of the different effects of input and output of O. Wong et al. [26] reported the application of combined evaluation of *δ* ^18^O and *δ* ^2^H fractionation factor (**FF**) in the passage from human plasma to urine and saliva. To our knowledge, no data are available on H and O stable isotope fractionation in dairy cattle from plasma to other fluid compartments (faecal water, urine, milk). Similarly, no data are available for a stable isotope approach to the evaluation of heat stress in livestock production, and particularly in dairy cattle, in which heat stress elicits a great adaptive reaction through changes in water metabolism, including their relationship with the changes due to the animal type (breed × reproductive stage). Considering the relationships between ingested water (free water + water in feed), water loss (faeces + urine + milk), and measurement of the isotope ratio in an easy-to-sample water-based component of the body (blood plasma), we propose an approach to detect possible variations in the isotope ratio in different body fluids on the basis of different homoeothermic adaptations.

## Materials and Methods

### Ethics statement

The sample collection protocol and animal care were in accordance with Directive 2010/63/EU of the European Parliament and the Council of 22 September 2010, on the protection of animals used for scientific purposes [27]. The animals were not subjected to any experimental procedures other than standard veterinary assessment through organic fluid sampling. This study was not presented to an animal research ethics committee because it was based on samples collected during routine veterinary assessment in both farms.

### Farm location and animal management

The study was carried out in two dairy farms, located in the Po valley, Lombardy Region: the first in Quinzano d’Oglio (45° 18’ 39” N, 10° 00’ 30” E, 51 m a.s.l., identified as **Farm BS**) and the second in Cremona (45° 08’ 00” N, 10° 02’ 00” E, 45 m a.s.l., identified as **Farm CR**).

**Fig 1:**
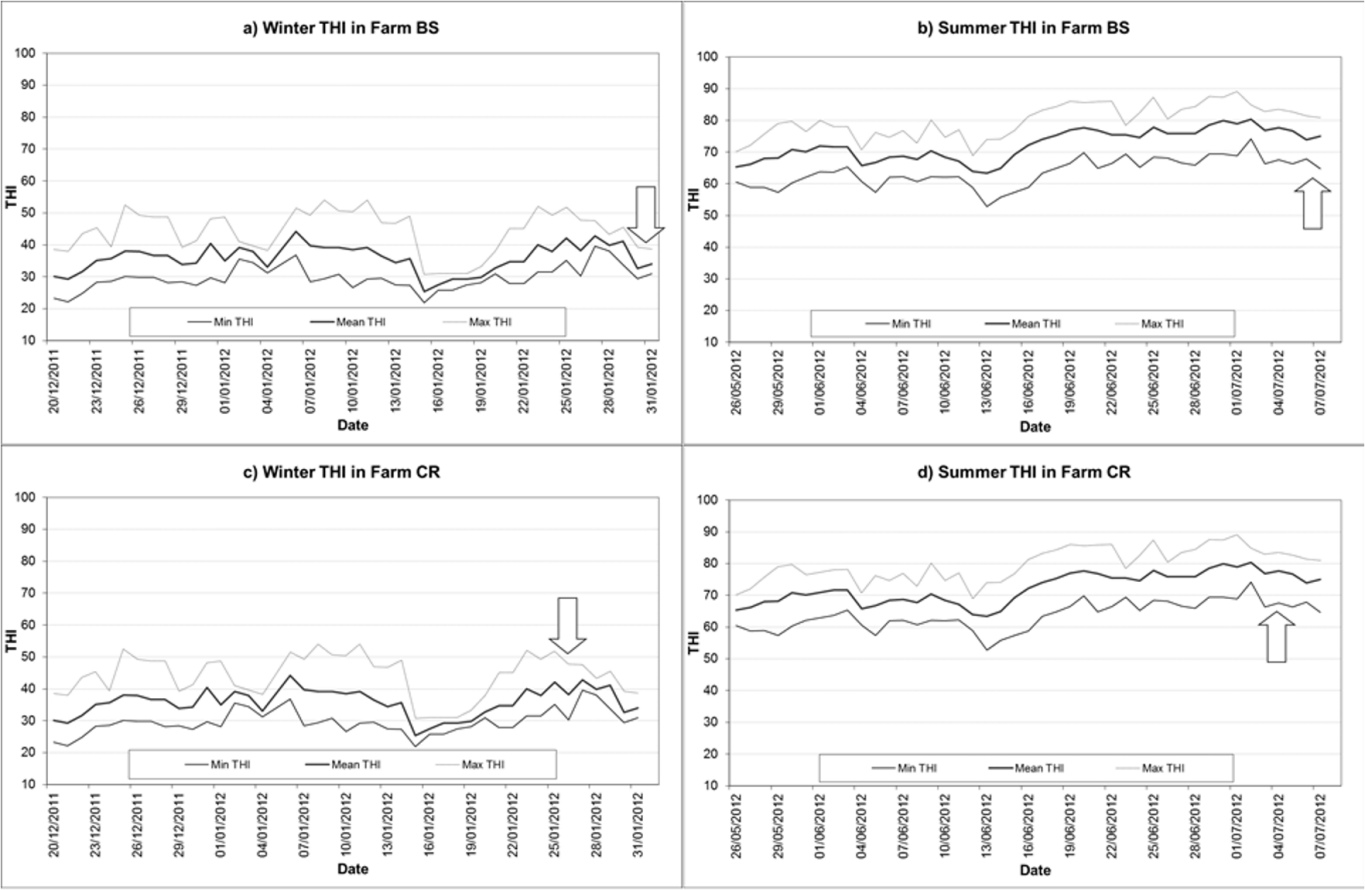
Recorded values for the Temperature Humidity Index (THI) in Farm BS and Farm CR before and at the time of controls. (a) Farm BS in winter. (b) Farm BS in summer. (c) Farm CR in winter. (d) Farm CR in summer. The arrows indicate the time of sampling for each farm in each season.

Three different reproductive stages of dairy cattle were considered: pregnant heifer (around 24–28 months of age), primiparous lactating cow, and pluriparous lactating cow. The same experimental scheme was practiced in comparable animals during two seasons: winter (Fig 1A and 1C) and summer (Fig 1B and 1D). The sampling time was planned to avoid the effect of the different water half-life (from 2.0 d for lactating cows to 7.5 d for non-lactating cows), starting from the beginning of steady climatic conditions between the different reproductive stages already highlighted [2]. For this reason, the sampling took place at least two weeks after relatively stable THI (Fig 1), in a supposedly steady state of body water turnover and, during heat stress, not in the phase of increasing body water pool to adapt to increased Ta [1, 28].

**Fig 2:**
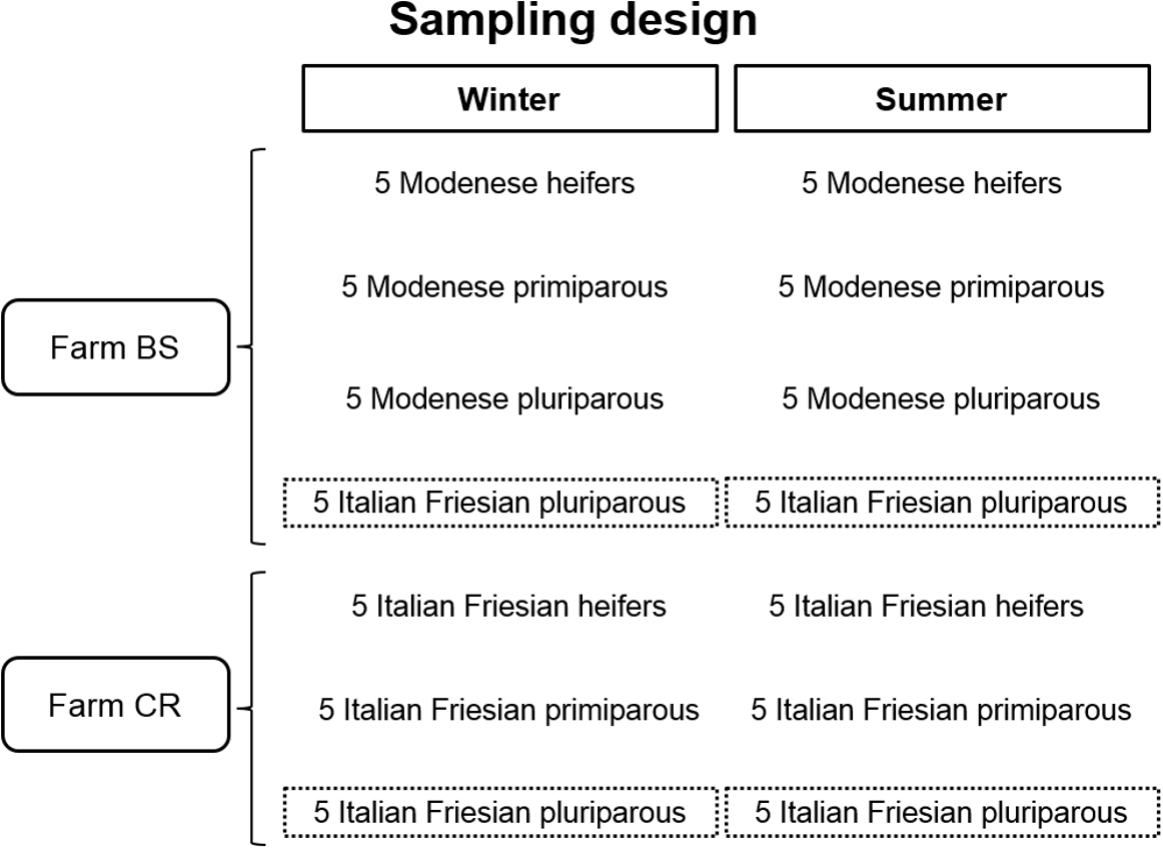
Sampling design. Italian Friesian pluriparous cows are highlighted in the dotted rectangles as the common type of cows tested in both farms.

The presence of pluriparous cows of two different breeds in Farm BS allowed comparison within the farm, highlighting possible differences between a cosmopolitan milk-specialised breed (Holstein type, represented by the Italian Friesian) and a local dual-purpose (milk and meat production) breed (Modenese) (see Fig 2 for sampling design).

The description of the monitored animals is shown in Tables 1 and 2 for Farm BS and Farm CR respectively. We had no previous measurements of the variability of these isotopic variables in cattle fluids; the number of replications in each randomised block was therefore based only on the number of possible subjects fitting within the previously defined category range: heifer, primiparous, and pluriparous cows.

**Table 1:** Descriptive statistics (mean ± sd) of the experimental groups in Farm BS.

Item	Season	Modenese heifers	Modenese primiparous	Modenese pluriparous	Italian Friesian pluriparous
Parity, n	Winter	-	1.00 ± 0.00	3.20 ± 0.45	3.20 ± 0.45
	Summer	-	1.00 ± 0.00	4.00 ± 1.73	3.60 ± 0.89
Age, mo	Winter	23.0 ± 0.5	32.3 ± 1.8	54.8 ± 4.0	60.0 ± 3.5
	Summer	26.1 ± 5.7	32.1 ± 6.9	64.4 ± 21.1	70.6 ± 6.6
Days from calving, d	Winter	-46 ± 11	115 ± 50	52 ± 48	97 ± 47
	Summer	-109 ± 72	76 ± 34	85 ± 26	159 ± 46
Daily milk yield, kg/d	Winter	-	12.0 ± 4.2	19.7 ±4.3	38.8 ± 6.1
	Summer	-	19.6 ± 3.8	22.4 ± 2.3	29.6 ± 7.1
Rectal temperature, °C	Summer	-	-	38.63 ± 0.19	38.73 ± 0.43
Breathing rate, n/30”	Summer	-	-	22.0 ± 1.0	30.8 ± 8.3

**Table 2:** Descriptive statistics (mean ± sd) of the experimental groups in Farm CR.

Item	Season	Italian Friesian heifers	Italian Friesian primiparous	Italian Friesian pluriparous
Parity, n	Winter	-	1.00 ± 0.00	3.40 ± 0.89
	Summer	-	1.00 ± 0.00	3.00 ± 0.71
Age, mo	Winter	26.1 ± 1.4	34.6 ± 8.2	66.0 ± 9.9
	Summer	30.4 ± 3.8	43.1 ± 14.4	64.3 ± 14.1
Days from calving, d	Winter	-47 ± 30	87 ± 33	94 ± 61
	Summer	-62 ± 23	104 ± 32	154 ± 23
Daily milk yield, kg/d	Winter	-	25.3 ± 7.4	35.2 ± 10.4
	Summer	-	31.2 ± 5.5	29.2 ± 6.7
Rectal temperature, °C	Summer	38.52 ± 0.13	38.60 ± 0.19	38.76 ± 0.57
Breathing rate, n/30”	Summer	26.4 ± 4.5	37.4 ± 5.5	35.0 ± 8.3

The two farms had similar housing systems for young non-lactating animals, with an open free-stall barn with cubicles in Farm BS, and an open free-stall barn with straw-bedding in Farm CR. The biggest difference in the housing systems in the farms was related to the barns for lactating cows. In Farm BS, the building was open (without perimeter walls) and without fans for ventilation (only natural). In Farm CR, the building was closed by perimeter walls (but with a paddock outside), and equipped with a fan-misting system set as follows: only fan, when inside the barn Ta > 20.5°C (4 min work + 15 min pause); uninterrupted operation, when Ta > 23.0°C; activation of misting lines when inside the barn Ta > 24.0°C (with increasing amounts at 25.0, 26.0, 27.5, and 28.5°C).

The diets in both farms were formulated according to NRC guidelines and were based on corn silage, grass hay and commercial concentrates [29]. Diets were based on similar corn silage quantities for lactating cows (25 kg in Farm BS and 26 kg in Farm CR) and pregnant heifers (7 kg in Farm BS and 8 kg in Farm CR). Dietary NDF, one of the most limiting factors for voluntary DMI [29], was similar in both farms for lactating cows (37.1% and 35.7% on DM basis for Farm BS and Farm CR respectively) and pregnant heifers (47.2% and 51.3% on DM basis for Farm BS and Farm CR respectively). The lactating cows (both primiparous and pluriparous) were fed in the same group within each farm, and lactating cows of the two breeds were fed in the same group in Farm BS.

### Sampling and data collection

Two sampling sessions were planned, trying to monitor the effect of a long period of low or high THI in winter and summer respectively. On the first day, samples of drinking water and corn silage were collected; on the second day, blood, urine and faeces samples were collected before feed distribution, and milk samples during the morning milking. These samples (blood, urine, faeces, milk) were collected as part of routine veterinary care. In Farm CR, the samples were collected by a veterinarian researcher from our group; in Farm BS, the samples were collected by the veterinarian of the farm, under the supervision of our veterinarian researcher.

Corn silage was frozen and the water contained was obtained by freeze-drying (Hetosicc CD 52–1, Heto, Birkerød, Denmark) at a temperature of -50°C for 5 days.

Blood samples were collected from the jugular vein using evacuated tubes (10 ml blood in a Li-heparin), cooled immediately after sampling and centrifuged in the laboratory within 2 hours of collection at 3000 g x for 20 min at 4°C. Plasma was divided into four aliquots (4 x 1.5 ml small plastic tubes) and stored at -20°C until assay.

Urine samples were collected at spontaneous emission, or just after mild vulvar stimulation, and a fraction was centrifuged at 500 × *g* for 5 min. Faeces samples were collected directly from the distal rectum, and a liquid fraction was obtained by centrifugation at 3000 × *g* for 15 min. Milk samples were defatted by 3 successive centrifugations at 1600 × *g* for 5 min. All samples were stored at -20°C until assay.

At the summer control, rectal temperature (using a digital thermometer) and breathing rate (through visual observation of the animal flank in two successive 30-sec periods) of pluriparous cows were recorded according to a previous study [30].

### Isotope ratio analysis

The isotope ratios of H (^2^H/^1^H) and O (^18^O/^16^O) in each sample were determined without any pre-treatment.

The ^18^O/^16^O of water was determined using an isotope ratio mass spectrometer (SIRA II, VG Isogas, Middlewich, UK or Isoprime, Manchester, UK) interfaced with an on-line automatic system that allows CO_2_/H_2_O equilibration (ISOPREP 18, VG Isotech, Middlewich, UK or Multiflow, Isoprime, Manchester, UK), according to the technique described by Epstein and Mayeda [31]. The ^2^H/^1^H ratio of water was analysed using the Isoprime instrument. 200 μl of water was pipetted into reaction vessels, where Hokko bead platinum catalysts (Isoprime) were placed to catalyse the equilibration of H_2_ with H_2_O. The vessels were then attached to an on-line automated equilibration system, filled with He containing 10% of H_2_ (Rivoira, Milano, Italy) and left to equilibrate for 4 hours at 40°C. The ^18^O/^16^O and ^2^H/^1^H ratios were expressed as delta per thousand (*δ* ^18^O‰ and *δ* ^2^H‰) as the deviation of the isotope ratio of the sample from the international reference standard VSMOW (Vienna Standard Mean Ocean Water). *δ* ^18^O‰ and *δ* ^2^H‰ values were measured and expressed relative to VSMOW reference water on a scale normalised by assigning the consensus values of -55.5‰ and −428 ‰ respectively to SLAP (Standard Light Antarctic Precipitation, IAEA-International Atomic Energy Agency, Vienna, Austria). Working in-house standards, prepared starting from tap water and calibrated against VSMOW and SLAP were periodically used to calibrate the measurements. After calibration, the tap water standard samples were stored at 4° and their isotopic values were checked using control charts. The analytical uncertainty (1 standard deviation) of *δ* ^2^H and *δ* ^18^O‰ measurements was < 2‰ and < 0.2‰ respectively.

### Calculations and statistical analysis

For each sample, deuterium excess (***d***) was calculated according to Dansgaard, as follows [32]:

$$d=\delta^{2}\text{H}-8\delta^{18}\text{O}$$ (1)

The isotope-fractionation factor (**FF**) was calculated for each passage from plasma to faeces, urine and milk within each animal at each sampling, according to the formula of Wong et al. as follows [26]:

$$\alpha =(\delta_{x}+1000)/(\delta_{plasma}+1000)$$ (2)

where *δ*_x_ and *δ*_plasma_ are the *δ* ^2^H or *δ* ^18^O values of the sample and plasma water respectively.

Global correlation coefficients (Pearson) between all the considered variables were calculated using the CORR procedure of SAS (version 9.3; SAS Institute Inc., Cary, NC).

Analysis of variance was conducted separately for each farm, to assess the fixed effects of season (winter, summer) and group (reproductive stage in Farm CR for Holstein type; reproductive stage for Modenese cows and lactating pluriparous Italian Friesian cows in Farm BS) using the GLM procedure of SAS (version 9.3; SAS Institute Inc., Cary, NC).

## Results

Table 3 shows the global correlation coefficients (Pearson) between stable isotope ratios in body fluids. The highest correlation coefficients were recorded between *δ* ^**18**^O in plasma and those in the other three compartments: milk, faeces, and urine (in decreasing order with values for all three fluids higher than 0.87). Intermediate correlation values were recorded between *δ* ^2^H and *δ* ^**18**^O in the same compartment, ranging from 0.768 in faecal water to 0.849 in milk. The lowest values were recorded for the correlations between *δ* ^2^H in different compartments (0.694–0.785).

**Table 3:** Global correlation coefficients (Pearson) for the δ-values of water stable isotopes in dairy cow fluids from both farms (for all the coefficients, P < 0.001).

Item	δ^2^H fecal water	δ^18^O fecal water	δ^2^H urine	δ^18^O urine	δ^2^H plasma	δ^18^O plasma	δ^2^H milk	δ^18^O milk
δ^2^H fecal water	1	0.7683	0.7129	0.7088	0.7521	0.8052	0.7567	0.7609
	δ^18^O fecal water	1	0.8214	**0.9219**	0.7676	**0.9548**	0.8077	**0.9312**
		δ^2^H urine	1	0.7695	0.7848	0.7739	0.6936	0.7127
			δ^18^O urine	1	0.7412	**0.9455**	0.8079	**0.8705**
				δ^2^H plasma	1	0.8000	0.7336	0.7710
					δ^18^O plasma	1	0.8084	**0.9602**
						δ^2^H milk	1	0.8493
							δ^18^O milk	1

Raw values of *δ* ^2^H and *δ* ^18^O in drinking water were very similar in the two farms, with values of -60 and -62‰ for *δ* ^2^H and -9.0 and -8.6‰ for *δ* ^18^O in winter, and of -65 and -61‰ for *δ* ^2^H, and -9.4 and -9.1‰ for *δ* ^18^O in summer in Farm BS and Farm CR respectively. The isotope values in the extracted water from corn silage, the main source of water from feed, were -45 and -47‰ for *δ* ^2^H and the same value (-4.5‰) for *δ* ^18^O for Farm BS and Farm CR respectively.

In both farms (BS and CR), the effect of the season on *δ* ^2^H and *δ* ^18^O was significant in all the fluids, with the sole exceptions of *δ* ^2^H in faecal water in Farm CR and the *δ* ^2^H in plasma of CR heifers (Tables 4 and 5). The effect of the group was significant for both isotopes in all the fluids, with the exception of milk in Farm CR, *δ* ^18^O in CR winter faecal water, and *δ* ^2^H in CR summer urine water and BS summer milk water. The interaction between season and group was significant in both farms only for *δ* ^18^O in faecal water, urine and plasma, but not in milk; this interaction was not significant for *δ* ^2^H, with the sole exception of milk in Farm BS (Tables 4 and 5). Numerically, the largest variation from winter to summer was recorded in urine *δ* ^2^H in both farms, particularly in the Italian Friesian breed. The effect of the group was particularly appreciable in Farm BS, where the Italian-Friesian breed had lower values compared with the other groups.

**Table 4:** Stable isotopes, deuterium excess (*d*), and fractionation factors (FF) in dairy cow fluids in Farm BS.

Item	Season			Group								P	
		Modenese heifers		Modenese primiparous		Modenese pluriparous		Italian Friesian pluriparous		s.e.m.	Season	Group	Season × Group
δ^2^H fecal water	Winter	-36	c	-42	b	-45	b	-49	a	0.42	< 0.0001	< 0.0001	-
	Summer	-31	b	-37	a	-38	a	-41	a				
		**		**		***		***					
δ^18^O fecal water	Winter	-4.7	c	-5.7	b	-5.9	ab	-6.3	a	0.06	< 0.0001	< 0.0001	0.023
	Summer	-1.7	c	-3.6	b	-3.9	ab	-4.3	a				
		***		***		***		***					
δ^2^H urine	Winter	-40	c	-45	bc	-44	b	-51	a	0.50	< 0.0001	< 0.0001	-
	Summer	-31	b	-39	a	-37	a	-41	a				
		***		*		***		***					
δ^18^O urine	Winter	-5.7	b	-6.4	a	-6.6	a	-6.6	a	0.07	< 0.0001	< 0.0001	0.045
	Summer	-2.8	b	-5.1	a	-4.4	a	-4.6	a				
		***		***		***		***					
δ^2^H plasma	Winter	-36	c	-42	b	-46	a	-47	a	0.41	< 0.0001	< 0.0001	-
	Summer	-32	c	-37	b	-38	ab	-41	a				
		*		**		***		***					
δ^18^O plasma	Winter	-4.7	c	-5.6	b	-5.7	b	-6.2	a	0.05	< 0.0001	< 0.0001	0.0002
	Summer	-1.5	c	-3.4	b	-3.5	b	-4.2	a				
		***		***		***		***					
δ^2^H milk	Winter			-46	b	-51	a	-52	a	0.44	< 0.0001	0.067	0.009
	Summer			-39		-39		-37					
				***		***		***					
δ^18^O milk	Winter			-6.3	b	-6.5	ab	-6.8	a	0.06	< 0.0001	0.0002	-
	Summer			-4.2	b	-4.2	b	-4.9	a				
				***		***		***					
Fecal water *d*	Winter	0.900		3.080		1.740		1.800		0.598	< 0.0001	0.009	0.048
	Summer	-16.975	a	-8.320	b	-7.500	b	-6.400	b				
		***		***		***		***					
Urine *d*	Winter	5.275	ab	6.700	ab	9.080	b	1.860	a	0.680	< 0.0001	0.015	-
	Summer	-8.600	a	0.950	b	-1.942	b	-3.760	ab				
		***				***		***					
Plasma *d*	Winter	2.275		3.060		-0.260		2.740		0.449	< 0.0001	0.0001	0.0001
	Summer	-19.640	a	-9.560	b	-10.040	b	-7.020	b				
		***		***		***		***					
Milk *d*	Winter			4.420		1.340		2.740		0.487	< 0.0001	0.005	0.003
	Summer			-5.320	a	-5.220	a	2.000	b				
				***		***							
Plasma—faeces													
^2^H FF	Winter	0.9991		0.9995		1.0009		0.9980		0.0005	-	-	-
	Summer	1.0020		0.9996		0.9999		0.9999					
^18^O FF	Winter	1.0000		0.9999		0.9998		0.9998		0.0001	-	-	-
	Summer	0.9999		0.9997		0.9996		0.9999					
Plasma—milk													
^2^H FF	Winter			0.9957		0.9953		0.9946		0.0005	<0.0001	-	0.046
	Summer			0.9980	a	0.9991	a	1.0035	b				
						*		***					
^18^O FF	Winter			0.9993		0.9992		0.9993		0.0001	-	-	-
	Summer			0.9992		0.9992		0.9992					
Plasma—urine													
^2^H FF	Winter	0.9954	a	0.9969	a	1.0020	b	0.9952	a	0.0005	-	0.038	-
	Summer	0.9994		0.9986		0.9999		0.9988					
^18^O FF	Winter	0.9990		0.9991		0.9990		0.9995		0.0001	-	0.024	-
	Summer	0.9988	a	0.9985	a	0.9993	ab	0.9996	b				

s.e.m. = standard error of the mean

Values within a row without a common letter differ for P < 0.05.

Values in the same column with asterisk differ for P < 0.05 (*), P < 0.01 (**) or P < 0.001 (***).

**Table 5:** Stable isotopes, deuterium excess (*d*), and fractionation factors (FF) in dairy cow fluids in Farm CR.

Item	Season			Group						P	
		Italian Friesian heifers		Italian Friesian primiparous		Italian Friesian pluriparous		s.e.m.	Season	Group	Season × Group
δ^2^H fecal water	Winter	-38	b	-44	a	-44	a	0.59	-	< 0.0001	-
	Summer	-36	b	-44	a	-43	a				
δ^18^O fecal water	Winter	-5.7		-6.1		-6.1		0.08	< 0.0001	< 0.0001	< 0.0001
	Summer	-2.8	b	-5.1	a	-5.0	a				
		***		**		***					
δ^2^H urine	Winter	-43	b	-47	a	-47	a	0.36	< 0.0001	0.006	-
	Summer	-39		-39		-40					
		**		***		***					
δ^18^O urine	Winter	-6.0	b	-6.7	a	-6.9	a	0.05	< 0.0001	< 0.0001	< 0.0001
	Summer	-3.9	b	-5.9	a	-6.0	a				
		***		***		***					
δ^2^H plasma	Winter	-40	b	-47	a	-46	a	0.45	0.0001	0.0001	-
	Summer	-38	b	-41	ab	-42	a				
				***		*					
δ^18^O plasma	Winter	-5.3	b	-6.2	a	-6.2	a	0.06	< 0.0001	< 0.0001	< 0.0001
	Summer	-2.7	b	-5.4	a	-5.4	a				
		***		**		***					
δ^2^H milk	Winter			-46		-47		0.43	0.0006	-	-
	Summer			-44		-43					
				*		**					
δ^18^O milk	Winter			-6.5		-6.7		0.07	< 0.0001	-	-
	Summer			-5.7		-5.7					
				*		***					
Fecal water *d*	Winter	7.320		4.000		4.008		0.607	< 0.0001	0.042	<0.0001
	Summer	-13.480	a	-3.220	b	-3.220	b				
		***		*		*					
Urine *d*	Winter	5.200		6.480		7.800		0.375	< 0.0001	< 0.0001	< 0.0001
	Summer	-7.660	a	8.020	b	7.520	b				
		***									
Plasma *d*	Winter	2.144		2.422		4.100		0.543	< 0.0001	< 0.0001	< 0.0001
	Summer	-16.120	a	2.460	b	1.620	b				
		***									
Milk *d*	Winter			5.620		5.980		0.532	0.008	-	-
	Summer			2.020		3.120					
				*							
Plasma—faeces											
^2^H FF	Winter	1.0016		1.0025		1.0012		0.0008	-	-	-
	Summer	1.0016		0.9969		0.9990					
				*							
^18^O FF	Winter	0.9995	a	1.0001	b	1.0002	b	0.0001	0.048	0.003	-
	Summer	0.9999	a	1.0003	ab	1.0005	b				
Plasma—milk											
^2^H FF	Winter			1.0007		0.9983		0.0006	-	-	-
	Summer			0.9970		0.9992					
^18^O FF	Winter			0.9997		0.9996		0.0001	-	-	-
	Summer			0.9997		0.9997					
Plasma—urine											
^2^H FF	Winter	0.9967		0.9994		0.9983		0.0005	0.011	0.086	-
	Summer	0.9992		1.0018		1.0015					
^18^O FF	Winter	0.9992		0.9994		0.9993		0.0001	-	0.003	0.089
	Summer	0.9988	a	0.9995	b	0.9994	b				
		*									

s.e.m. = standard error of the mean

Values within a row without a common letter differ for P < 0.05.

Values in the same column with asterisk differ for P < 0.05 (*), P < 0.01 (**) or P < 0.001 (***).

**Fig 3:**
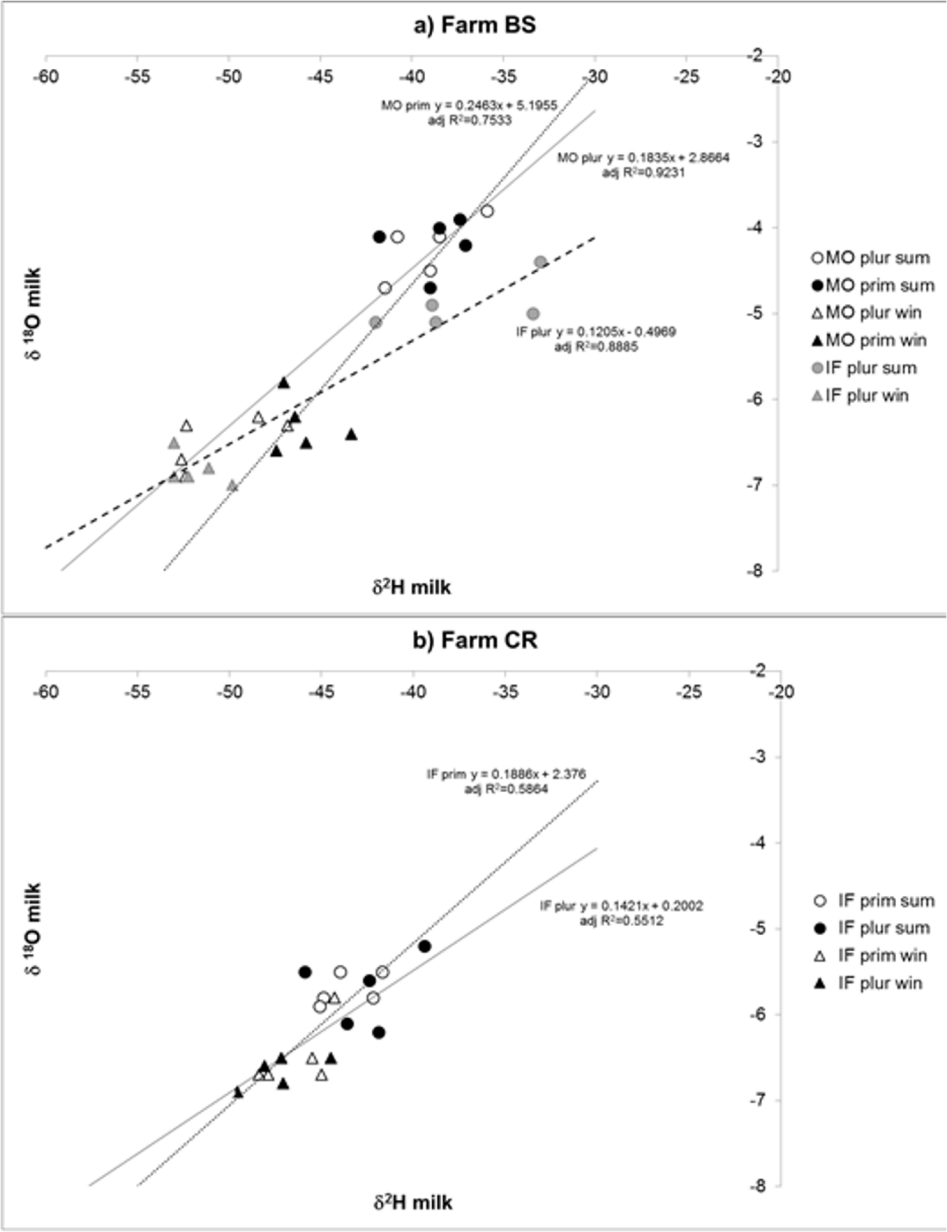
Biplot obtained from *δ* ^2^H and *δ* ^18^O in milk from the two pluriparous and Modenese primiparous experimental groups in both seasons in Farm BS, (a). **Biplot obtained from *δ*** ^**2**^ **H and *δ*** ^**18**^ **O in milk from primiparous and pluriparous Italian Friesian cows in Farm CR, (b).** MO plur sum = Modenese pluriparous during summer; MO prim sum = Modenese primiparous during summer; MO plur win = Modenese pluriparous during winter; MO prim win = Modenese primiparous during winter; IF plur sum = Italian Friesian pluriparous during summer; IF plur win = Italian Friesian pluriparous during winter; IF prim sum = Italian Friesian primiparous during summer; IF prim win = Italian Friesian primiparous during winter.

Fig 3 shows the plot of milk isotopes in pluriparous cows of the two breeds in Farm BS (Fig 3A) and in primiparous and pluriparous Italian Friesian cows in Farm CR (Fig 3B). In Farm BS, there was a clear separation between milk produced in winter and that produced in summer. This separation was more extensive than separation due to parity (among Modenese cows) or breed (among pluriparous cows). Within summer samples, there was also separation between milk from Italian Friesian and from Modenese cows. The extent of separation due to the season was less pronounced in Farm CR, where a winter sample from a primiparous cow appeared to be confusable with those obtained from both primiparous and pluriparous cows during summer. None of summer samples was confusable with winter milk.

Deuterium excess (Tables 4 and 5) was affected (highly significantly) by season in all the fluids analysed and in both farms, except in urine for Modenese primiparous cows, in milk for Friesian pluriparous cows at the BS farm, in urine and plasma in primiparous cows, and in urine, plasma and milk in pluriparous cows at the CR farm. The effect of group was significant, albeit to a variable extent, in all fluids at both farms, with the exception of winter faecal water, plasma and milk in Farm BS, and all the winter fluids from Farm CR. The interaction between group and season was significant for all the fluids except milk in Farm CR and urine in Farm BS. The season affected FF little in both farms (Tables 4 and 5). The most significant relationship was for *δ* ^2^H in the plasma-milk passage in Farm BS, with higher FF in summer. The only FFs affected by group in both farms were those from plasma to urine for both *δ* ^2^H and *δ* ^**18**^O. The other common result in both farms was the lack of effect of the considered factors on *δ* ^**18**^O FF from plasma to milk.

## Discussion

### Correlations between H and O stable isotopes in body fluids

This is the first time that correlations between water isotope measurements in bovine body fluids have been reported. While on one hand the general (and always significant) correlation between these values seems obvious, the most interesting result is the range of these coefficients (Table 3). Indeed, the correlation coefficients between *δ* ^2^H in the different body fluids ranged between 0.6936 (urine-milk) and 0.7848 (urine-plasma), but the correlation coefficients between *δ* ^18^O were markedly higher, ranging between 0.8705 (urine-milk) and 0.9602 (plasma-milk). This different pattern suggests that O isotopes have fewer points of fractionation or may be less subject to interference, such as that related to metabolic requirements (with O uptake), than H isotopes. However, it must be considered that the uncertainty of measurement is 10 times greater for *δ* ^2^H than for *δ* ^18^O and this could negatively affect the correlations between H isotopes in the different fluids.

The high correlation coefficients between *δ* ^**18**^O in different compartments suggest that this isotope was poorly affected by measurement uncertainty (as previously reported), and by local metabolism in the tissues touched by the fluid: rumen and gut for faecal water; kidney for urine; udder for milk. The intermediate correlation values recorded between *δ* ^2^H and *δ* ^**18**^O within compartments are attributable to the different extent of fractionation for the two isotopes (see also the differences in *d*).

### Water partition in body compartments according to environmental temperature, animal breed and reproductive stage

For a better understanding of the results presented here, we could compare them with three different studies conducted under different experimental conditions. All these studies demonstrate an increase in free water intake and water evaporation with heat stress, together with possible increased water content in the cow’s body [13, 28, 33]. This increase is mainly due to increased water content in the rumen as a result of an increased water turnover rate [34]. By evaluating the absolute amount of water input and output for the cows during the trials, we argue that there is a shift in water turnover from urinary excretion, a theoretically non-isotope fractionating path, to evaporation, a possible isotope-fractionating path. The increase in evaporated water ranged from 8% to 64% per m^2^ of body surface area, as calculated by Richards [28], and from 26.5% to 93.1% in the study of McDowell et al. [33].

In this study, the isotope values in drinking water and in water extracted from corn silage were similar in both farms and season. This observation permitted us to exclude possible interferences due to variations in diet composition. The increase in both isotope *δ* values observed in all body fluids during the summer period is explainable by a shift from not-fractionating paths (faecal water, urine, and milk) to fractionating paths (breath and transcutaneous water vapour) [18]. The summer increase in *δ*-values could be considered the result of fractionation within the animal driven by water metabolism in response to environmental changes. The data confirmed that water partitioning is strongly affected by temperature increases. This is due to the primary function played by water in cattle thermoregulation [3], which allows for the transfer of a large amount of heat in a small “carrier” volume [2]. Such changes in water partitioning in animal thermoregulation could be considered a typical example of the gain and loss of mass with different isotope composition, as reported by Fry [17]. In the event of external variation (e.g. climate change) which may occur to an animal in steady conditions, fractionation is due to water vaporisation and a simultaneous increase in drinking water intake which is characterised by a different isotopic composition. The new water equilibrium necessary to address thermoregulation could be demonstrated by a new balance between H and O isotopes.

Milk production is a leading factor in water intake and water turnover. Therefore, the differing aptitudes for milk production (a consequence of the genetic selection criteria adopted in the two breeds), and changes in water partitioning across the different physiological stages, could be considered possible sources of differences in isotopic composition between the two breeds and/or the two reproductive stages (pregnant heifer and lactating cow). The Modenese is a dual-purpose breed characterized by a white coat colour. It produce meat and medium-low quantities of milk (on average, around the half of that recorded in the Italian Friesian), this way suggesting a quite different metabolic rate considering the little difference in body size between the two breeds [35]. As consequence, the genetic background selected for this different specialization, with high metabolic rate in high milk yielding cattle, may be responsible for a poor sweating response [15, 16]. We recently reported significant differences in metabolism between Modenese and Italian Friesian during the periparturient period [35]. The different body water turnover in adult lactating cattle and young pregnant non-lactating heifers was part of the reason for the higher isotopic *δ* in the latter, in both breeds. Indeed, the shift in the equilibrium towards values more distant from those of drinking water could be a consequence of low water turnover in heifers. In contrast, lactating cattle have a higher turnover due to milk production. The difference between the two reproductive stages, heifers and lactating cows, was consistent with the difference between seasons: the higher the role of evaporative losses (compared to urine and milk), the higher the *δ* values in the fluids.

Comparing the two breeds in Farm BS, the main difference between pluriparous cows was in faecal water, and this could be considered a possible effect of different water intake to compensate for higher body water turnover in Italian Friesian cows as compared to Modenese cows. In both farms, *δ* ^18^O in faecal water was higher in summer than in winter, but *δ* ^2^H in faecal water was higher in summer only in Farm BS. Understanding the meaning of these results for ruminants may at first seem difficult. A first explanation may be related to the consistency of these changes in other body fluids across the various groups. Indeed, the isotopic *δ* values of faecal water were very different from those of drinking water in both seasons, but nearer to those of blood plasma values. If we consider the important role of salivary secretion and its daily production, which in dairy cattle can vary from 100 to 250 l/d [5], together with data reported by Woodford et al. [9], the magnitude of water recycling by saliva may be 3–6 times the amount of daily water intake as drinking water. This proportion may account for the values of isotope *δ* in faecal water, which may be more affected by salivary water than the *δ* ^2^H of drinking water. Salivary water derives from recycling fluids from several body compartments by the circulatory system. As a result, its isotope equilibrium reflects that of plasma, as has been determined in humans [26]. Considering the values in milk, it is clear from the plot of *δ* ^2^H and *δ* ^18^O (Fig 3) for pluriparous cows of the two breeds in Farm BS that winter milk is not separable according to breed, whereas summer milk is. This distinguishability in summer conditions was probably the result of a different response of the two breeds to heat stress, with seemingly higher fractionation in Modenese cows. Additionally, separation according to season was more evident in this farm. The separation according to season was more evident in this farm, but the observations in both environments apparently gave no explanation. However, a possible hypothesis could be based on the different characteristics of the buildings on the farms. The Farm BS was without perimeter walls (totally open barn), while the Farm CR was with perimeter walls (albeit with two wide facing openings in the barn). We did not measure the actual air speed close to the cows in the two buildings; the open barn in Farm BS could have favoured higher body vapour elimination, this way increasing the extent of fractionation as compared to Farm CR. In Farm CR, with the sole exception of a milk sample from a primiparous cow in winter (which seemed not to be different from summer milk), a separation based on the season seems quite plausible. In both farms it was not possible to separate primiparous from pluriparous cows. The apparent overlapping of primiparous and pluriparous water isotopes in milk suggests that other factors affected this feature more than parity per se.

Fig 3, which includes regression curves within each breed at each physiological stage, highlights the different behaviour of the water isotope equilibrium in different seasons. By comparing Fig 3A and 3B, we can see that there was a change in the slope of the regression line between primiparous and pluriparous cows within each breed. In Fig 3A, we can also see that Italian Friesian pluriparous cows differed from Modenese pluriparous cows in the intercept of the regression curve, which was positioned, in the observed range, below that of Modenese cows. Together, these regressions highlight the different extent of fractionation in water isotopes for each group in response to the change in season. Further research on H and O in the solid component of milk (namely casein), and in cheese, would be useful for traceability and forensic purposes.

These findings would also be useful for studying climate during the life of cattle, when mineralised tissue is available. Indeed, oxygen and hydrogen atoms in the drinking water and the consequential equilibrium between body water pools can also affect the composition of tissues such as hair and tooth enamel [36].

The results from this study on body fluids agree with the extent of a summer-winter differentiation of 2–3 ‰ in *δ* ^**18**^O and 2–10 ‰ *δ* ^2^H that was evidenced in cattle hair by Auerswald et al. [37]. At the same time, this seasonal difference was similar to that reported by D’Ambrosia et al. [38] which studied the stable isotope pattern in equid teeth according to the dentition pattern in a fall or a spring reproductive season.

In light of the results by Podlesak et al. [36] concerning the relationships between *δ* ^**18**^O in body water and tooth enamel, we can speculate that our results confirm the current methods applied in paleoclimatic reconstructions [37, 38] without regard to the age or reproductive state of the animal remains. However, specific research needs to be oriented on possible factors that may interfere with the incorporation of isotopic differences from body fluids to mineralized tissues.

It would be harder, from current results, to understand if breed differences could correctly be attributed in paleobiological studies. This is because our practicable observations for this kind of comparison were obtained only in 5 pluriparous Italian Friesian and 5 pluriparous Modenese for each season only within Farm BS. Therefore, a wider study than the current one will be necessary to answer the question concerning the detectability of breed differences.

### Deuterium excess: meaning of this new item in a biological system

The concept of deuterium excess is widely adopted in geology and geophysical sciences [32], but has not been employed in biological studies until now. The *d*-value is used as an index for non-equilibrium in environmental, geochemical and geophysical studies [32]. It is based on the assumption of fractionation of H and O according to their physical features within the water molecule. Its use is uncommon in biological studies on water metabolism. We introduced this approach in an attempt to quantify and understand whether different isotope fractionation occurs for H and O atoms. From these preliminary results, *δ* ^2^H and *δ* ^18^O combined with deuterium excess *d* would seem to give the same degree of information about the effects of heat stress as the two separate items. We tested *d* as a possible tool to refine and possibly increase the information deriving from the analytical results on *δ* ^2^H and *δ* ^**18**^O, considering the study by Wong et al. [26] who reported an isotopic fractionation factor from plasma to respiratory evaporation that was much lower for ^2^H than for ^18^O. This was also the case in this study, with a shift towards lower (and often negative) values highlighting the relatively lower fractionation of H. We do not know the reason for this result, but it was evident that it behaves according to the season (always numerically lower in summer), and reproductive stage (always numerically lower in non-lactating heifers during summer). These differences are linked to reduced metabolic activity in the animal, due to heat stress and the lack of milk production (an activity associated with high metabolic expenditure).

We have no specific reference for isotope equilibrium in evaporative water from cattle, but it seems reasonable to surmise fractionation similar to that reported by Wong et al. [26] in humans, where there is a very low value for *δ* ^2^H.

### Water isotope fractionation in mammalian physiology

FF is the least comparable of the parameters investigated here, because of the limited availability of data in the relevant literature [26]. The few significant effects on FF are consistent with high correlations of isotopic *δ* between body fluids, confirming the physiological trend of the body to equilibrate water isotopes across the different compartments. Only the kidney seemed to be a possible significant fractionation point, as highlighted by analysis of plasma-urine FF in both farms, particularly for the group effect.

Analysis of isotopic FF in animal science is less diffuse than analysis of *δ* ^2^H and *δ* ^18^O. From the results presented here, isotopic FF from blood plasma to the other biological fluids was less consistent in the farms.

## Conclusions

To our knowledge, this is the first paper that has approached the detection of changes in water metabolism in heat-stressed dairy cattle through the analysis of *δ* ^2^H and *δ* ^**18**^O in body fluids. Our first conclusion is therefore that this approach would seem to be interesting and promising. In particular, the matters considered made it possible to discriminate between body fluids collected in different seasons. This not only confirms previous observations on milk by other researchers, but would also seem to be a good starting point for planning further research on the use of water stable isotopes as markers of heat stress in dairy cattle. This was confirmed by the effect of reproductive stage on the stable isotope equilibrium highlighted in this paper as the consequence of different water turnover in lactating and non-lactating cows.

## Supporting Information

S1 Dataset Dataset of the body fluids measurements. Legend: sid = sample identification code; pid = animal identification code; season = sampling season (winter, summer); breed = animal breed (modenese = Modenese, itfriesian = Italian Friesian); farm (farmcr, farmbs); group = reproductive stage (IFHEIF = Italian Friesian heifer, IFPRIM = Italian Friesian primiparous, IFPLUR = Italian Friesian pluriparous, MOHEIF = Modenese heifer, MOPRIM = Modenese primiparous, MOPLUR = Modenese pluriparous); parity = number of calving at the sampling time; birthday = date of birth; age_days = age (days); age_months = age (months); sampling_date = date of sampling; d2Hfec = δ^2^H in fecal water; d18Ofec = δ^18^O in fecal water; d2Hur = δ^2^H in urine; d18Our = δ^18^O in urine; d2Hpl = δ^2^H in blood plasma; d18Opl = δ^18^O in blood plasma; DIM = days from calving; d2Hmilk = δ^2^H in milk; d18Omilk = δ^18^O in milk; ffplfeh = fractionation factor from blood plasma to fecal water for H; ffplfeo = fractionation factor from blood plasma to fecal water for O; ffplurh = fractionation factor from blood plasma to urine for H; ffpluro = fractionation factor from blood plasma to urine for O; ffplmih = fractionation factor from blood plasma to milk for H; ffplmio = fractionation factor from blood plasma to milk for O; defec = deuterium excess in fecal water; deuri = deuterium excess in urine; = depla = deuterium excess in blood plasma; demilk = deuterium excess in milk.
(XLS)
